# Alterations in the *CTRB2* gene and response to chemotherapy in pancreatic cancer

**DOI:** 10.1371/journal.pone.0343022

**Published:** 2026-02-19

**Authors:** Khalid Jazieh, Erin E. Carlson, Zafar Siddiqui, Katelyn E. Connelly, Jun Zhong, Jason W. Hoskins, Kari G. Rabe, MAYO-RGC Project Generation, Regeneron Genetics Center, Ann L. Oberg, Hugues Sicotte, Ryan M. Carr, Laufey T. Amundadottir, Samuel O. Antwi

**Affiliations:** 1 Division of Medical Oncology, Mayo Clinic, Rochester, Minnesota, United States of America; 2 Department of Quantitative Health Sciences, Division of Clinical Trials and Biostatistics, Mayo Clinic, Rochester, Minnesota, United States of America; 3 Department of Internal Medicine, Mayo Clinic, Rochester, Minnesota, United States of America; 4 Laboratory of Translational Genomics, Division of Cancer Epidemiology and Genetics, National Cancer Institute, National Institutes of Health, Bethesda, Maryland, United States of America; 5 Department of Quantitative Health Sciences, Computational Biology, Mayo Clinic, Rochester, Minnesota, United States of America; 6 Department of Quantitative Health Sciences, Division of Epidemiology, Mayo Clinic, Jacksonville, Florida, United States of America; Universita degli Studi di Roma Tor Vergata, ITALY

## Abstract

**Background:**

Pancreatic ductal adenocarcinoma (PDAC) remains a highly fatal malignancy due partly to treatment resistance in many patients. We previously identified a functional germline deletion overlapping exon 6 of *CTRB2* (CTRB2ex6) at a PDAC genome-wide risk locus on chr16q23.1. This variant leads to a nonfunctional truncated chymotrypsin protein that accumulates intracellularly and induces endoplasmic reticulum stress. Here, we performed a retrospective study to determine whether CTRB2ex6 deletions are associated with response to chemotherapy, time to cancer progression, or overall survival (OS) in PDAC patients.

**Methods:**

The study included *CTRB2* genotype data from two independent PDAC cohorts (Cohort 1: n = 633; Cohort 2: n = 3,896). We examined associations between CTRB2ex6 deletion status and OS and time-to-progression (TTP) using Cox proportional hazard modeling. TTP was also evaluated in a subset of chemotherapy-treated patients in Cohort 1 (n = 263) to determine the impact of CTRB2ex6 deletion status on chemotherapy response.

**Results:**

CTRB2ex6 deletions were found in 20% of patients in both cohorts combined (19% in Cohort 1, and 21% in Cohort 2). No significant difference was observed in OS by *CTRB2* deletion status in either cohort (Cohort 1: HR = 0.95, *p* = 0.60; Cohort 2: HR = 1.04, *p* = 0.43). Among chemotherapy-treated patients in Cohort 1, *CTRB2* deletion carriers had a longer median TTP (20.4 vs. 12 months), though this was not statistically significant (*p* = 0.49). Homozygous deletion carriers had the longest TTP (70 months).

**Discussion:**

No clinical impact on chemotherapy response or OS was observed by *CTRB2* deletion status. Further studies are needed to identify reliable biomarkers of therapy response in PDAC.

## Introduction

Pancreatic ductal adenocarcinoma (PDAC), the third leading cause of cancer-related deaths in the US, has a five-year survival rate of only 8% and remains a major challenge for both patients and clinicians [1]. A primary reason for this is that the majority of patients are diagnosed with unresectable disease—only 16% of PDACs are diagnosed at the localized stage [1]. Further, PDAC is resistant to systemic therapy, which is the mainstay of management for metastatic disease. Even the most efficacious combinations of chemotherapy agents demonstrated short median overall survival (OS) for metastatic PDAC, with 11.1 months OS for FOLFIRINOX [2], 8.5 months for gemcitabine + nab-paclitaxel [3], and 11.1 months for NALIRIFOX [4].

There are several proposed mechanisms underlying chemoresistance in PDAC. These include cellular factors, such as expression of certain nucleoside transporters [5], enzyme activity [6], and epithelial-mesenchymal transition [7]. There are also elements of the tumor microenvironment that make PDAC resilient during treatment, including desmoplasia [8] and intratumoral hypoxia [9]. Despite this, there is very little clinically relevant information that could inform chemotherapy selection. Pathogenic germline variants in DNA repair genes, such as *BRCA1*, *BRCA2*, and *PALB2* have been shown to increase sensitivity of PDAC to platinum agents [10]. Hence, clinical guidelines recommend the use of FOLFIRINOX or gemcitabine + cisplatin in patients who carry pathogenic variants in certain DNA repair genes [11].

Our group previously fine-mapped a PDAC genome-wide association study (GWAS) signal on chr16q23.1 and identified a germline deletion variant (584 bp) that underlies risk at this locus [12]. The deletion (risk-increasing allele) overlaps exon 6 of the *CTRB2* gene and leads to a premature stop codon in exon 7 of *CTRB2*. The resulting truncated chymotrypsinogen B2 protein lacks chymotrypsin activity and accumulates intracellularly in the endoplasmic reticulum (ER) where it leads to the activation of the Unfolded Protein Response (UPR) [12], a pro-survival mechanism triggered by accumulation of unfolded or misfolded proteins [13]. Mouse models carrying exon 6 deletion in the *CTRB2* gene (the ortholog of human *CTRB2*) recapitulated the *in vitro* results showing dramatic ER stress, downregulation of the pancreatic acinar program and upregulation of inflammatory pathways *in vivo* [14]. In addition to its function to increase ER protein folding capacity and metabolic reprogramming, both highly important for actively growing cells, the UPR has been shown to increase drug efflux and cancer stem cell expansion under chemotherapy [15–18].

Here, we hypothesized that the activation of the UPR could negatively affect chemotherapy response in carriers of *CTRB2* exon 6 deletion variants and lead to earlier resistance to therapy and potentially shorten survival. To test this hypothesis, we performed association analysis of genetic variation in *CTRB2* and response to chemotherapy and OS using data from two independent cohorts.

## Materials and methods

### Study design and participants

We performed a retrospective study, leveraging existing genetic data on PDAC patients recruited into the Mayo Clinic Biospecimen Resource for Pancreas Research registry. The design and methods used for recruitment into the pancreas research registry have been published [19–21]. Briefly, an ultra-rapid case finding process was used to recruit patients into the registry with approximately 86% of patients recruited within 30 days of cancer diagnosis, with an overall median of two weeks between first contact and recruitment. The PDAC patients in the pancreas research registry from which the study samples were drawn had pathological (~95%) or radiographical confirmation of PDAC. These patients donated peripheral blood samples at recruitment, from which leukocyte DNA was extracted for genotyping. Genotyping was performed in two batches (Cohorts 1 and 2). Cohort 1 comprised patients who had blood samples collected between 10/03/2007 and 12/31/2008, and were genotyped as part of the Pancreatic Cancer Cohort (PanScan) and the Pancreatic Cancer Case Control (PanC4) consortia GWASs [22,23]. Cohort 2 comprised PDAC patients who had blood samples collected from 01/01/2009–12/31/2020 and were included in a collaborative genotyping project between the Mayo Clinic and the Regeneron Genetics Center (RGC), as described below. Data were accessed from 01/15/2025–09/02/2025. The PDAC patients for this study were restricted to those included in the two genotyping projects that had follow-up data allowing ascertainment of their survival status. All patients provided informed written consent, including consent for the use of their data and biospecimen for future studies. The present study was reviewed and approved by the Mayo Clinic Institutional Review Board (IRB #: 08–008031) and is compliant with the ethical guidelines of the Helsinki and Istanbul Declarations.

### Covariate data

The PDAC patients completed identical risk factor questionnaires that solicited various information, including demographics (e.g., age, sex, race/ethnicity, education level), personal and family health history, and self-reported usual adult weight and height. Clinical data, including PDAC stage at diagnosis, surgical, radiation and/or chemotherapy treatments received, information on disease progression, and overall survival were abstracted from patient medical records by trained abstractors. Body mass index (BMI) was calculated from self-reported weight and height in kg/m^2^. Additional information on data collection has been published [19,20,24].

### Genotyping and bioinformatics processing

The genotyping procedures used for Cohort 1 have been described extensively in previous PanScan and PanC4 publications [12,22]. Thus, here, we only describe the genotyping procedures for Cohort 2 that was performed by RGC using a genotyping-by-sequencing (GxS) assay [25,26], and the bioinformatics processes used on the resulting genetic data. Briefly, the RGC sequencing assay is a hybrid capture consisting of the Twist Comprehensive Exome custom capture and the Twist Diversity single nucleotide polymorphism (SNP) panel. This results in an assay that expands the standard exome capture with a genotyping “backbone” region tagging common variants for genome-wide scans. These backbone regions were sequenced at lower depth and underwent extensive post-GxS processing to improve genotyping quality based on linkage disequilibrium and population allele frequencies. The genotyped reads were aligned to the human reference genome (GRCh38) using BWA-mem and extensive quality control checks were performed, including exclusion of samples with sex discordance, poor exome coverage, contamination >5%, and duplicated samples. The genotyped variants restricted to the design sites of the Diversity SNP panel were refined and phased using GLIMPSE v1.0.0 [27]. Single-sample GLIMPSE refined variants were aggregated with GLnexus v1.4.3 using the pre-configured GxS setting into a multi-sample project-level VCF (pVCF), which was converted to analytic data formats using PLINK 1.9. The resulting genetic data were processed through the Mayo Clinic Genotype quality control pipelines that involved exclusion of SNPs based on poor genotyping call rate (< 0.95), minor allele frequency (MAF, < 0.05), and violation of the Hardy-Weinberg Equilibrium (*p* < 1 x 10^−6^). Participant samples were excluded if they had a large proportion of missing genotypes (>5%) or had abnormal heterozygosity (<70% on ≥2 chromosomes). Relatedness was examined using PLINK and PRIMUS to estimate identical-by-descent with removal of closely related samples (PI_HAT > 0.1875) or >25000 related samples (PI_HAT > 0.08). All samples with predicted first- and second-degree relationships were removed to generate a data set consisting of unrelated patient samples for further analysis.

The Cohort 2 *CTRB2* genotypes were computed from the exome sequencing data using programs available on github.com/sicotteh/CTRB2_584del. The program’s cutoffs were calibrated using a subset of 605 samples from Cohort 1 which were genotyped for the deletion using polymerase chain reaction (PCR), 9 of which were homozygous deletion (both copies of the allele were absent) and 106 of which were heterozygous for the deletion (only one copy of the allele was absent). Briefly, the software uses SAMtools 1.18 [28], to count reads spanning a 584 bp deletion encompassing exon 6 (reads mapping to chr16:75204400–75205700 on GRCh38), read pairs with anomalously large insert size (supporting the deletion) and read pairs with normal insert size (not supporting the deletion). The total coverage also includes reads with anomalous mapping (appear to be large insertion but caused by a germline *CTRB1* inversion) as an additional indicator of total coverage [12,29]. Genotypes were assigned as follows: if only reads supporting the deletion are found (and none supporting the exon 6 sequence), genotype is classified as 1/1; if no reads supporting the deletion were found, genotypes were 0/0, otherwise genotype was 0/1. Additional details on the bioinformatics processing of the data are provided in the **S1 File**.

### Endpoints

The primary endpoints of the study were OS and time-to-progression (TTP), and the secondary endpoint was chemoresistance. OS was calculated from the date of PDAC diagnosis to the date of death (event), with participants censored at the date last known to be alive in the event of loss to follow-up or date of last follow-up in December 2023, whichever came first. We calculated TTP from date of first PDAC-directed treatment to date of documented disease progression (event), with participants censored based on date of occurrence of any competing risk condition (death, receipt of surgery for non-progressive disease or receipt of another chemotherapy for non-progression reasons), or date last known to be alive in the event of loss to follow-up or date of last follow-up in December 2023, whichever came first. Resistance to chemotherapy was defined as having imaging or laboratory confirmed progression of the PDAC warranting a change in treatment plan (first change in treatment due to progressive disease).

### Statistical analysis

Participants’ characteristics were summarized using medians (range) for continuous variables, and frequencies and proportions for categorical variables. CTRB2ex6 deletion status was defined as having at least one deletion (heterozygous 0/1 or homozygous 1/1). Separately, we examined CTRB2ex6 deletion status using a three-level variable (homozygous 0/0, heterozygous 0/1, and homozygous 1/1). We used Kaplan-Meier survival curves to describe survival probabilities by CTRB2ex6 deletion status and calculated median survival in years. TTP was used to determine the impact of CTRB2ex6 deletion on response to treatment in a subset of patients who received chemotherapy. The associations of CTRB2ex6 deletion status with OS and TTP were examined using Cox proportional hazard models to calculate hazard ratios (HRs) and 95% confidence intervals (CIs), adjusting for age, sex, tumor stage at diagnosis, and type II diabetes mellitus. Additionally, we examined the association between CTRB2ex6 deletion status and TTP by tumor stage (localized, locally advanced, or metastatic), and exploratory analysis by type of first-line chemotherapy received (gemcitabine-based or 5-fluorouracil [5-FU]-based chemotherapy), adjusting for age, sex, and type II diabetes mellitus. All statistical tests were two-sided, and *p* < 0.05 were considered statistically significant. Analyses were performed using SAS 9.4 (SAS Institute, Cary, NC).

## Results

Descriptive statistics of the participants are shown in Table 1. The study sample included 633 PDAC patients in Cohort 1 (with a subset of 263 chemotherapy-treated patients), and 3,896 PDAC patients in Cohort 2. The patients in Cohort 2 included a greater proportion of women and individuals with a personal history of type II diabetes as compared to Cohort 1, while the patients in Cohort 1 were more frequently diagnosed with localized PDAC and had a higher self-reported BMI than patients in Cohort 2. There was no significant difference in CTRB2ex6 deletion carrier status between the PDAC patients in Cohort 1 (19%) and Cohort 2 (21%). In the subset of Cohort 1 patients treated with chemotherapy, 20% were CTRB2ex6 deletion carriers. Median overall follow-up time was 11.4 months for Cohort 1, 14.2 months for Cohort 2, and 13.9 months for both cohorts combined.

**Table 1:** Descriptive statistics of the PDAC cases included in the study.

	Cohort 1^a^		Cohort 2^b^
	Chemotherapy treated (n = 263)	Overall (n = 633)	Overall (n = 3896)
Sex, Female, n (%)	110 (41.8%)	286 (45.2%)	2231 (57.3%)
Age, median [range]	66 (28-86)	67 (28-90)	66 (20-92)
Stage at Dx, n (%) Localized Locally Advanced Metastatic Unknown	99 (39.0) 86 (33.8) 69 (27.2) 9	197 (32.0) 220 (35.8) 198 (32.2) 18	1120 (28.9) 1474 (38.0) 1280 (33.0) 22
Diabetes, n (%) No Yes Unknown	185 (72.8) 69 (27.2) 9	437 (71.6) 173 (28.4) 23	2124 (65.0) 1143 (35.0) 629
BMI, median [range] Unknown	27.6 (16.5-53.4) 0	27.4 (15.3-53.4) 0	22.7 (12.7-64.2) 22
*CTRB2* carrier/*genotype*, n (%) Non-carrier (0/0) Deletion carrier (0/1, 1/1) *Heterozygous (0/1)* *Homozygous (1/1)*	209 (79.5) 54 (20.5) *48 (18.2)* *6 (2.3)*	514 (81.2) 119 (18.8) *110 (17.4)* *9 (1.4)*	3091 (79.3) 805 (20.7) *733 (18.8)* *72 (1.8)*
First-line chemotherapy, n (%)			–
Gemcitabine only	73 (27.8)	–	
5-FU only	103 (39.2)		
Gemcitabine + 5-FU	20 (7.6)		
Gemcitabine + other	45 (17.1)		
5-FU + other	3 (1.1)		
Other chemotherapy	19 (7.2)		

^a^Patients included in previous genome-wide association studies conducted in collaboration with the Pancreatic Cancer Cohort Consortium (PanScan) and the Pancreatic Cancer Case Control (PanC4) consortia

^b^Patients included on genotype by sequencing project performed in collaboration with the Regeneron Genetics Center

Abbreviations: Dx., diagnosis; PDAC, pancreatic ductal adenocarcinoma; 5-FU, 5-fluorouracil

### Overall survival

**Fig 1:**
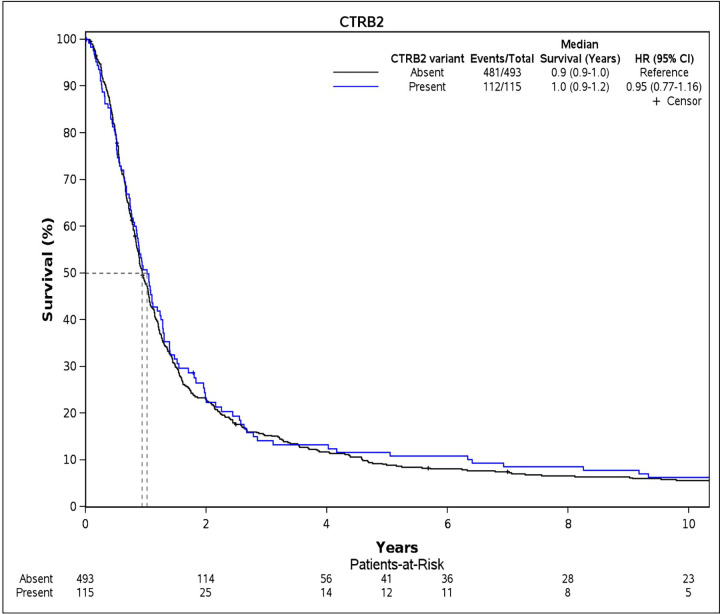
Kaplan-Meier survival curves for overall survival by CTRB2 deletion-carrier status in Cohort 1. Cohort 1 comprises pancreatic ductal adenocarcinoma patients included in previous genome-wide association studies conducted in collaboration with the Pancreatic Cancer Cohort Consortium (PanScan) and the Pancreatic Cancer Case Control (PanC4) consortia (n = 608, after removing patients with missing covariate data).

**Fig 2:**
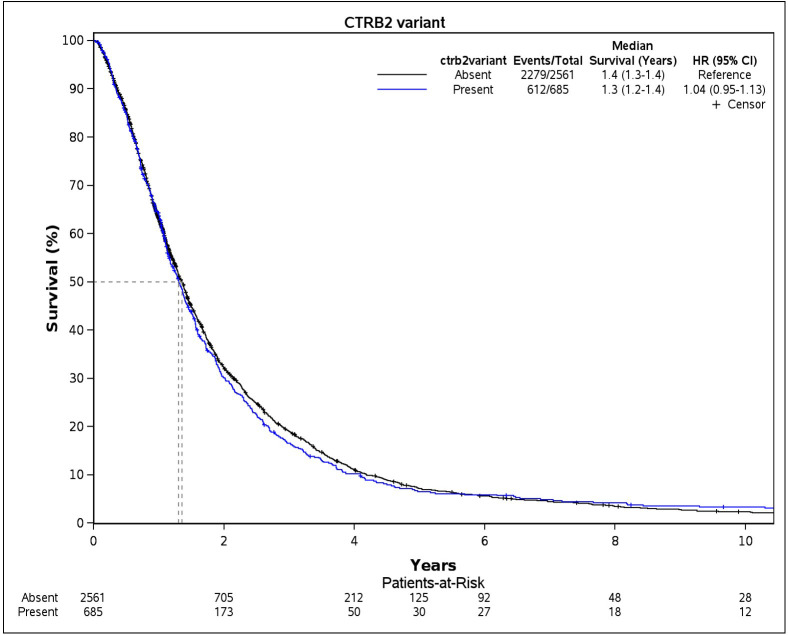
Kaplan-Meier survival curves for overall survival by CTRB2 deletion-carrier status in Cohort 2. Cohort 2 comprises pancreatic ductal adenocarcinoma patients included in a genotyping by sequencing project performed in collaboration with the Regeneron Genetics Center (n = 3246 after removing patients with missing information).

We examined survival probabilities by CTRB2ex6 deletion status using dominant modeling (any deletion carriers vs. non-carriers). In Cohort 1, median OS for CTRB2ex6 deletion carriers was 10.8 months compared to 12 months for non-carriers (Fig 1). In Cohort 2, the median OS among carriers of CTRB2ex6 deletion was 15.6 months compared with 16.8 months for non-carriers (Fig 2). We further explored survival probabilities by CTRB2ex6 genotype, and found that in Cohort 1, the nine homozygous deletion carriers (1/1) had a median OS of 31.2 months, while the 106 heterozygous deletion carriers (0/1) had a median OS of 10.8 months (S1 Fig). In Cohort 2, the 67 homozygous deletion carriers had a median OS of 15.6 months, and the 618 heterozygous deletion carriers had a median OS of 15.6 months (S2 Fig). Additionally, results from the Cox proportional hazard models did not show an association between CTRB2ex6 deletion carrier status and OS in Cohort 1 (HR = 0.95, 95% CI:.0.77–1.16, *p*-value = 0.60) or Cohort 2 (HR = 1.04, 95% CI: 0.95–1.13, *p*-value = 0.43).

We further examined the association between CTRB2ex6 deletion status and overall survival by tumor stage at diagnosis (Table 2). No significant associations were observed within any stage category in Cohort 1, Cohort 2, or in a combined analysis. In the combined cohort, the HR (95% CI) for localized, locally advanced, and metastatic disease were 1.01 (0.86–1.19), 1.06 (0.93–1.21), and 0.99 (0.86–1.13), respectively.

**Table 2:** Association between *CTRB2* exon 6 deletion status and overall survival by tumor stage of patients with PDAC.

*CTRB2* exon 6 deletion	N	Median OS (95% CI) months	HR (95% CI)^a^	*P*-value
**Cohort 1**				
Localized				
Absent	176	24.0 (19.2-36.0)	1.00 (ref)	
Present	29	31.2 (27.6-76.8)	0.74 (0.49-1.13)	0.15
Locally advanced				
Absent	166	10.8 (9.6-10.8)	1.00 (ref)	
Present	44	9.6 (8.4-13.2)	0.97 (0.69-1.36)	0.86
Metastatic				
Absent	151	8.4 (6.0-8.4)	1.00 (ref)	
Present	42	7.2 (6.0-10.8)	1.18 (0.83-1.68)	0.37
**Cohort 2**				
Localized				
Absent	776	24.0 (22.8-27.6)	1.00 (ref)	
Present	203	22.8 (19.2-25.2)	1.07 (0.90-1.27)	0.46
Locally advanced				
Absent	981	16.8 (15.6-18.0)	1.00 (ref)	
Present	267	15.6 (13.2-18.0)	1.11 (0.96-1.28)	0.17
Metastatic				
Absent	804	10.8 (9.6-10.8)	1.00 (ref)	
Present	215	10.8 (9.6-13.2)	0.96 (0.82-1.12)	0.61
**Cohorts 1 and 2 combined**				
Localized				
Absent	952	24.0 (22.8-26.4)	1.00 (ref)	
Present	232	24.0 (20.4-28.8)	1.01 (0.86-1.19)	0.88
Locally advanced				
Absent	1147	15.6 (14.4-16.8)	1.00 (ref)	
Present	311	14.4 (13.2-16.8)	1.06 (0.93-1.21)	0.37
Metastatic				
Absent	955	9.6 (9.6-10.8)	1.00 (ref)	
Present	257	10.8 (8.4-12.0)	0.99 (0.86-1.13)	0.84

^a^Adjusted for age, sex, and diabetes status in the cohort 1 and 2 analyses, and with additional adjustment for cohort in the combined analysis.

Abbreviations: CI, confidence interval; HR, hazard ratio; OS, overall survival; PDAC, pancreatic ductal adenocarcinoma

### Time-to-progression

**Fig 3:**
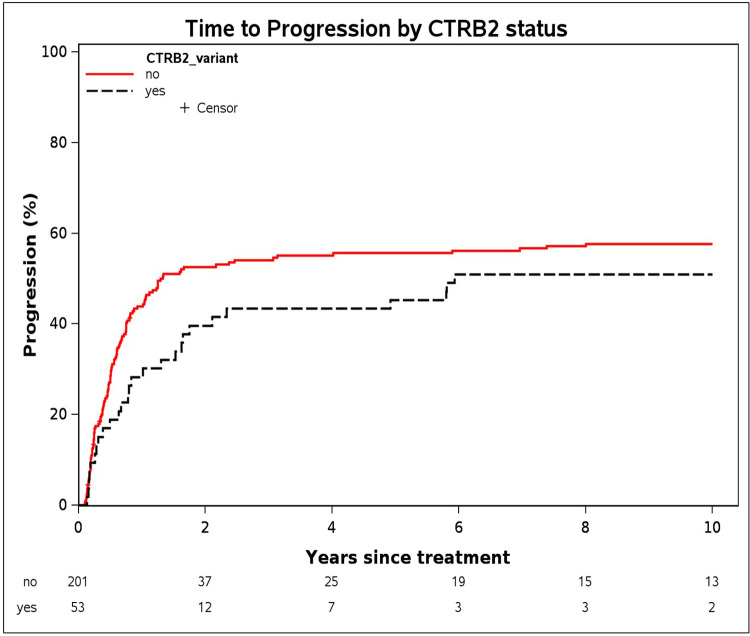
Time-to-progression based on CTRB2 deletion carrier status in a subset of pancreatic adenocarcinoma patients in Cohort 1 who were treated with chemotherapy. Cohort 1 comprises pancreatic ductal adenocarcinoma patients included in previous genome-wide association studies conducted in collaboration with the Pancreatic Cancer Cohort Consortium (PanScan) and the Pancreatic Cancer Case Control (PanC4) consortia (n = 254 after removing patients with missing information).

Of the 263 chemotherapy treated patients in Cohort 1, 148 (56%) patients developed progressive disease, while the remaining 115 patients did not have evidence of disease progression. CTRB2ex6 deletion carriers had a longer median TTP of 20.4 months compared to 13.2 months for non-carriers based on dominant modeling (Fig 3). When CTRB2ex6 deletion carriers were analyzed by genotype, the six homozygous deletion carriers had a median TTP of 69.6 months, and the remaining 47 heterozygous deletion carriers had a median TTP of 18 months (S3 Fig). Results from the Cox models did not show an association between CTRB2ex6 deletion carrier status and TTP based on dominant modeling (HR = 0.78 95% CI: 0.52–1.19, p-value = 0.42). We also conducted exploratory subgroup analyses by chemotherapy type (5-FU-based vs. gemcitabine-based) to evaluate a potential predictive role of the CTRB2ex6 deletion. No significant associations were observed among deletion carriers treated with 5-FU-based regimens (HR = 0.66, 95% CI: 0.35–1.27) or gemcitabine-based regimens (HR = 0.80, 95% CI: 0.33–1.97) (S1 Table).

## Discussion

In this retrospective analysis of data from two independent cohorts, we found CTRB2ex6 deletions in 20% of PDAC patients in both cohorts combined (19% in Cohort 1, and 21% in Cohort 2). Although CTRB2ex6 deletion increases UPR activity *in vitro* and *in vivo*, suggesting a potential role in chemoresistance, we did not observe an association with chemoresistance in either cohort. TTP for patients in Cohort 1 who received chemotherapy was not significantly different based on CTRB2ex6 deletion carrier status. The carriers of a CTRB2ex6 deletion had longer median TTP than those that did not, and patients with homozygous deletions had the longest TTP. This contradicted our hypothesis that CTRB2ex6 deletions contribute to chemoresistance. In both cohorts, there was no significant difference in OS based on CTRB2ex6 deletion carrier status. We did not find significant differences in TTP and deletion status by tumor stage at diagnosis. Overall, we observed no statistically significant association between CTRB2ex6 deletion status and OS, TTP, or chemotherapy response in PDAC patients; however, there was suggestive evidence of a trend toward longer TTP among deletion carriers.

Although CTRB2ex6 deletions were not significantly associated with PDAC prognosis in the present study, this finding may reflect the complex and heterogeneous nature of PDAC, in which prognosis is driven by a combination of tumor aggressiveness, microenvironment, metabolic reprograming, and therapeutic resistance mechanisms [30–32]. Additionally, the limited number of CTRB2ex6 deletion cases may have reduced statistical power to detect modest prognostic differences. Notably, we observed a suggestive, non-statistically significant trend toward longer TTP among patients with CTRB2ex6 deletions. While this finding should be interpreted with caution, it raises the possibility that CTRB2ex6 deletion status may be associated with differential sensitivity to chemotherapy or delayed tumor progression. *CTRB2* encodes a digestive enzyme precursor and is expressed in the pancreas; alterations in this gene could potentially reflect broader changes in acinar function, tumor differentiation, or tumor-stroma interactions that influence treatment response dynamics [33,34]. Future studies in larger, independent cohorts and functional investigations are needed to clarify the biological and clinical relevance of the observed trend.

Our study has several strengths and limitations. Strengths include the use of data from two independent cohorts. To our knowledge, this is the first study to evaluate the potential clinical impact of CTRB2ex6 deletions on PDAC patient outcomes, informed by our prior study that implicated deletions in CTRB2ex6 in PDAC development [12]. Limitations include the retrospective abstraction of clinical data, which is less precise than a prospective data collection. Most patients in Cohort 1 did not have sufficient treatment information to be included in the TTP analysis due to the nature of our practice as a tertiary care center, where patients come to seek second opinions on cancer diagnosis, prognosis and treatment plans, or only present to our center for surgery and might have received their chemotherapy treatments at their home institution. This resulted in a limited statistical power of our TTP analysis. The study population might not be fully representative of broader or more diverse populations or other patients within other healthcare settings, which may limit generalizability. We also did not have treatment data on the Cohort 2 patients. Further, Cohort 1 included patients diagnosed until 2008, which was before the approval of FOLFIRINOX (2011) [2] and gemcitabine + nab-paclitaxel (2013) [3] for treatment of metastatic PDAC. Another limitation of this study is the potential for selection bias resulting from the exclusion of participants with incomplete data. Because of limited information on individuals who were excluded from the analysis, we could not compare their characteristics with those of the participants included in the study regarding key demographic, clinical, or risk factor information. Thus, the direction of this potential bias remains uncertain and could have influenced the findings either toward or away from the null. This limitation should be considered in interpreting the results.

In summary, we did not find an association between CTRB2ex6 deletion status and OS or TTP. We did not observe an adverse impact of CTRB2ex6 deletions on chemotherapy response, or differences between deletion status and TTP by tumor stage at diagnosis. However, we observed a trend toward longer TTP among deletion carriers, highlighting a potentially relevant signal that warrants validation in larger cohorts. Because very little data exists on genetics-based treatment for PDAC to guide therapy selection, beyond what is known about *BRCA*1/2, further investigations into predictors of response to treatment in PDAC could have a significant impact on patient care. It would also be helpful to include genomic and transcriptomics data collection as part of prospective trials to advance our understanding of the complex biology of PDAC and identify genomic vulnerabilities to overcome treatment resistance for this frequently fatal cancer.

## Supporting information

S1 File Provides analysis code, and detailed information on the genomic coordinates and mapping of the CTRB2 gene. (PDF)

S1 Fig Kaplan-Meier survival curves for overall survival by CTRB2 deletion-carrier status based on three-level genotypes in Cohort 1. Cohort 1 comprises pancreatic ductal adenocarcinoma patients included in previous genome-wide association studies conducted in collaboration with the Pancreatic Cancer Cohort Consortium (PanScan) and the Pancreatic Cancer Case Control (PanC4) consortia (n = 608 after removing patients with missing information).
(JPEG)

S2 Fig Kaplan-Meier survival curves for overall survival by CTRB2 deletion-carrier status based on three-level genotypes in Cohort 2. Cohort 2 comprises pancreatic ductal adenocarcinoma patients included in a genotyping by sequencing project performed in collaboration with the Regeneron Genetics Center (n = 3246 after removing patients with missing information).
(JPEG)

S3 Fig Time-to-progression based on CTRB2 deletion carrier status based on three-level genotypes in a subset of pancreatic ductal adenocarcinoma patients in Cohort 1 who were treated with chemotherapy. Cohort 1 comprises pancreatic ductal adenocarcinoma patients included in previous genome-wide association studies conducted in collaboration with the Pancreatic Cancer Cohort Consortium (PanScan) and the Pancreatic Cancer Case Control (PanC4) consortia (n = 254 after removing patients with missing information).
(JPEG)

S1 Table Association between CTRB2ex6 deletion status and time to progression (TTP) by treatment type among patients with PDAC. (DOCX)

## Abbreviations

CTRB2ex6: exon-6 of the chymotrypsinogen B2 gene
ER: endoplasmic reticulum
GWAS: genome-wide association study
OS: overall survival
PDAC: pancreatic ductal adenocarcinoma
RCG: Regeneron Genetics Center
TTP: time-to-progression
UPR: Unfolded Protein Response.
