# Supplementary material for: Alterations in the CTRB2 gene and response to chemotherapy in pancreatic cancer
Source: PLoS One. 2026 Feb 19;21(2):e0343022. doi: 10.1371/journal.pone.0343022 (PMC12919833; doi:10.1371/journal.pone.0343022)
Supplement: S1 File — (PDF) [file pone.0343022.s001.pdf]

Supplementary Methods

Analytic code: The source code for detecting the *CTRB2* 584bp deletion in exome data is available here: https://github.com/sicotteh/CTRB2_584del

Background: The 584 bp *CTRB2* deletion has been described in detail by Jermusyk *et al*. [1]. The deletion removes the following region, encompassing exon 6 of the *CTRB2* gene:

- chr16: 75,238,615–75,239,199 (GRCh37) +/- 5 bp
- chr16: 75,204,717–75,205,301 (GRCh38) +/- 5bp

The deletion on *CTRB2* is identical to a region on a nearby gene, *CTRB1,* which likely arose from a gene duplication. A fraction of the general population harbors an inversion of the *CTRB1* locus relative to the *CTRB2* locus, and this is the allele represented in the reference human genome (both GRCh37 [hg19] and GRCh38 [hg38]). The extent of the identical region on GRCh38 (save for 1 bp) is:

- chr16: 75204677 75206214* *CTRB2* (mismatch at 75205641T)
- chr16: *75222761 75224283 *CTRB1* (mismatch at 75223320(rev(A)=T))

with the deletion being close to the left end of the *CTRB2* gene as in **Supplementary Methods Figure 1**.

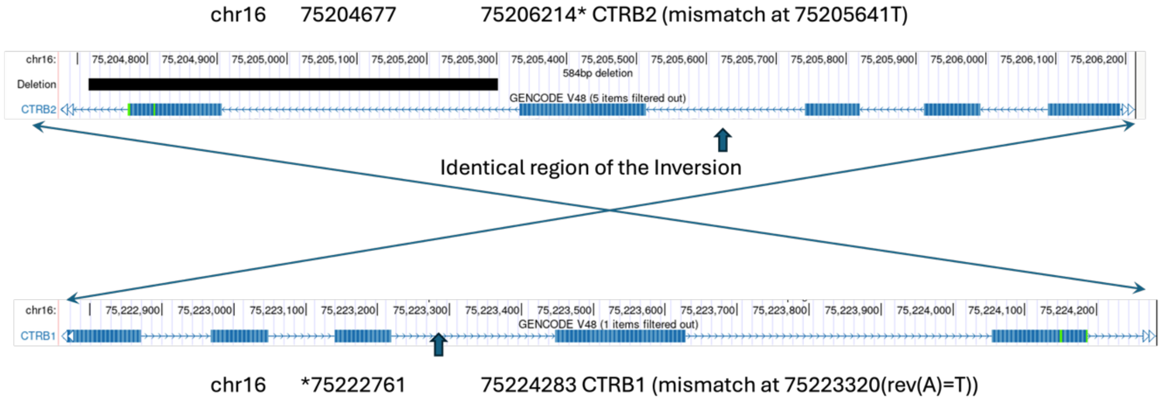

Two haplotypes in the Population. One with the inversion (chosen as the reference genome) and the second without the inversion. Only this latter Haplotype can include a deletion in CTRB2.

Supplementary Methods Figure 1. The *CTRB2* gene (exons 6,5,4,3,2) is identical to the *CTRB1* region (same exons), except that on the reference genome, the represented haplotype has the *CTRB1* gene inverted. The region shown is identical base for base (except for 1 base, indicated by vertical arrows). Note the inversion extends past this identical region. The 584bp deletion is shown overlapping from after exon 5 and ending past (left side) exon 6.

The 584bp deletion is only present on the haplotype that does not include the inversion (the CTRB1 is not inverted as in the reference genome). The strategy to detect the deletion from reads is detailed in **Supplementary Methods Figure 2** (Haplotype with the deletion) and Supplementary Figure 3 (Haplotype without the deletion). The key to calling that deletion is that reads to the left of the deletion extends outside the inversion region and thus can be <u>uniquely mapped</u>, while reads on the other side of the deletion (or on the right side of the deletion start) will map up to two locations.

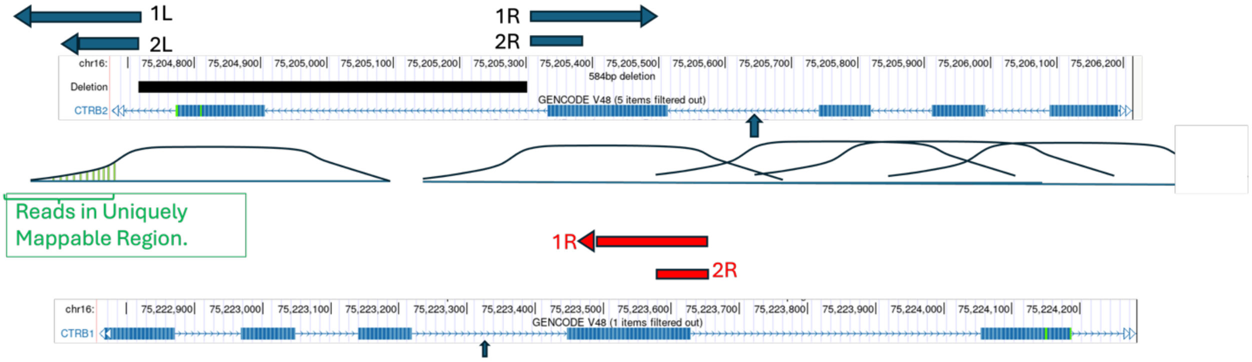

Supplementary Methods Figure 2. Mapping of the deletion. We detected the deletion using paired end reads. The deletion happens where we can expect reads from the exome capture to span each side of the deletion (shown by idealized coverage curves). We can expect pair end reads with anomalously long insert sise (read1) or split read (read 2). The 1L and 2L reads uniquely map to the edge of the inversion region on *CTRB2* because they are outside the homology inversion region, but the other end will mostly map to the other side of the deletion and – not as frequently – map to the *CTRB1* region. Please note that with our short 76 reads, there were no split reads in our dataset, so we did not use them.

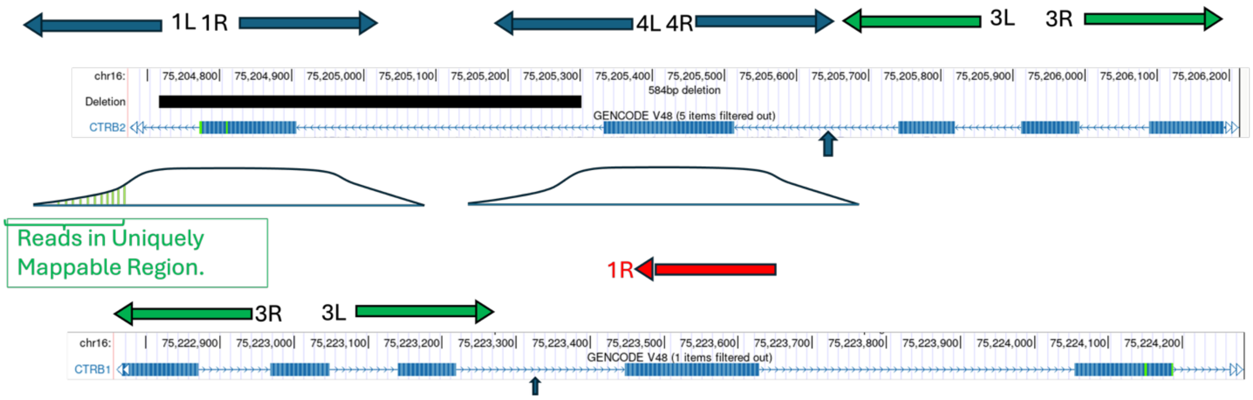

Supplementary Methods Figure 3: Mapping without the deletion. Without the deletion, the “1” style paired end reads can be uniquely mapped because there is a small part of the 1L that maps to a unique region. All the “3” style reads fully inside the region and will map to both the *CTRB1* or *CTRB2* region with equal mapping quality (except perhaps if 1 member of a pair of reads overlap the one mismatching site – like read 4). For the exon detection method, we only count reads of type 1.

Using SAMtools 1.18 [2], we counted read pairs spanning a 584 bp deletion in *CTRB2* [1]encompassing exon 5 (reads mapping to chr16:75204400-75205700 on GRCh38), counting read pairs with anomalously large insert size (supporting the deletion) and read pairs with normal insert size (not supporting the deletion). The total coverage also includes read pairs with anomalous mapping (appear to be large insertion but caused by a germline *CTRB1* inversion) as an additional indicator of total coverage [1, 3]. Genotypes were assigned as follows: if only reads supporting the deletion are found (and none supporting the exon 6 sequence), genotype is classified as 1/1; if no reads supporting the deletion were found, genotypes were 0/0, otherwise genotype was 0/1. The program’s cutoffs were calibrated from a subset of 605 samples which were also assayed for the deletion using PCR, 9 of which were homozygous deletion and 106 of which were heterozygous for the deletion.

References:

1. Jermusyk A, Zhong J, Connelly KE, Gordon N, Perera S, Abdolalizadeh E, et al. A 584 bp deletion in CTRB2 inhibits chymotrypsin B2 activity and secretion and confers risk of pancreatic cancer. Am J Hum Genet. 2021;108(10):1852-65. Epub 20210923. doi: 10.1016/j.ajhg.2021.09.002. PubMed PMID: 34559995; PubMed Central PMCID: PMCPMC8546220.
2. Danecek P, Bonfield JK, Liddle J, Marshall J, Ohan V, Pollard MO, et al. Twelve years of SAMtools and BCFtools. Gigascience. 2021;10(2). doi: 10.1093/gigascience/giab008. PubMed PMID: 33590861; PubMed Central PMCID: PMCPMC7931819.
3. Pang AW, Migita O, Macdonald JR, Feuk L, Scherer SW. Mechanisms of formation of structural variation in a fully sequenced human genome. Hum Mutat. 2013;34(2):345-54. Epub 20121119. doi: 10.1002/humu.22240. PubMed PMID: 23086744.
