# Supplementary figures and images for: Alterations in the CTRB2 gene and response to chemotherapy in pancreatic cancer
Source: PLoS One. 2026 Feb 19;21(2):e0343022. doi: 10.1371/journal.pone.0343022 (PMC12919833; doi:10.1371/journal.pone.0343022)

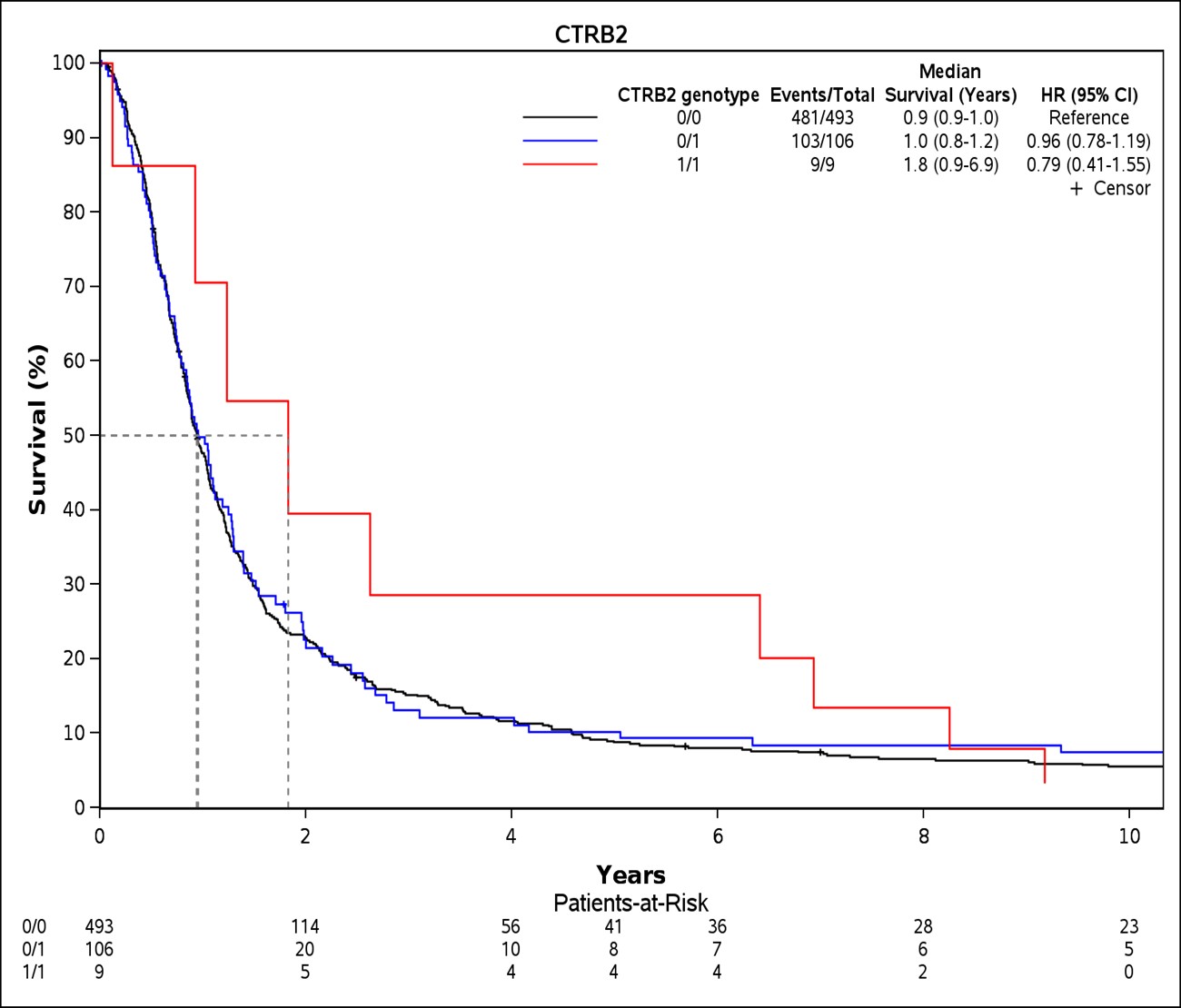

Supplement: S1 Fig — Cohort 1 comprises pancreatic ductal adenocarcinoma patients included in previous genome-wide association studies conducted in collaboration with the Pancreatic Cancer Cohort Consortium (PanScan) and the Pancreatic Cancer Case Control (PanC4) consortia (n = 608 after removing patients with missing information). (JPEG) [file pone.0343022.s002.jpeg]

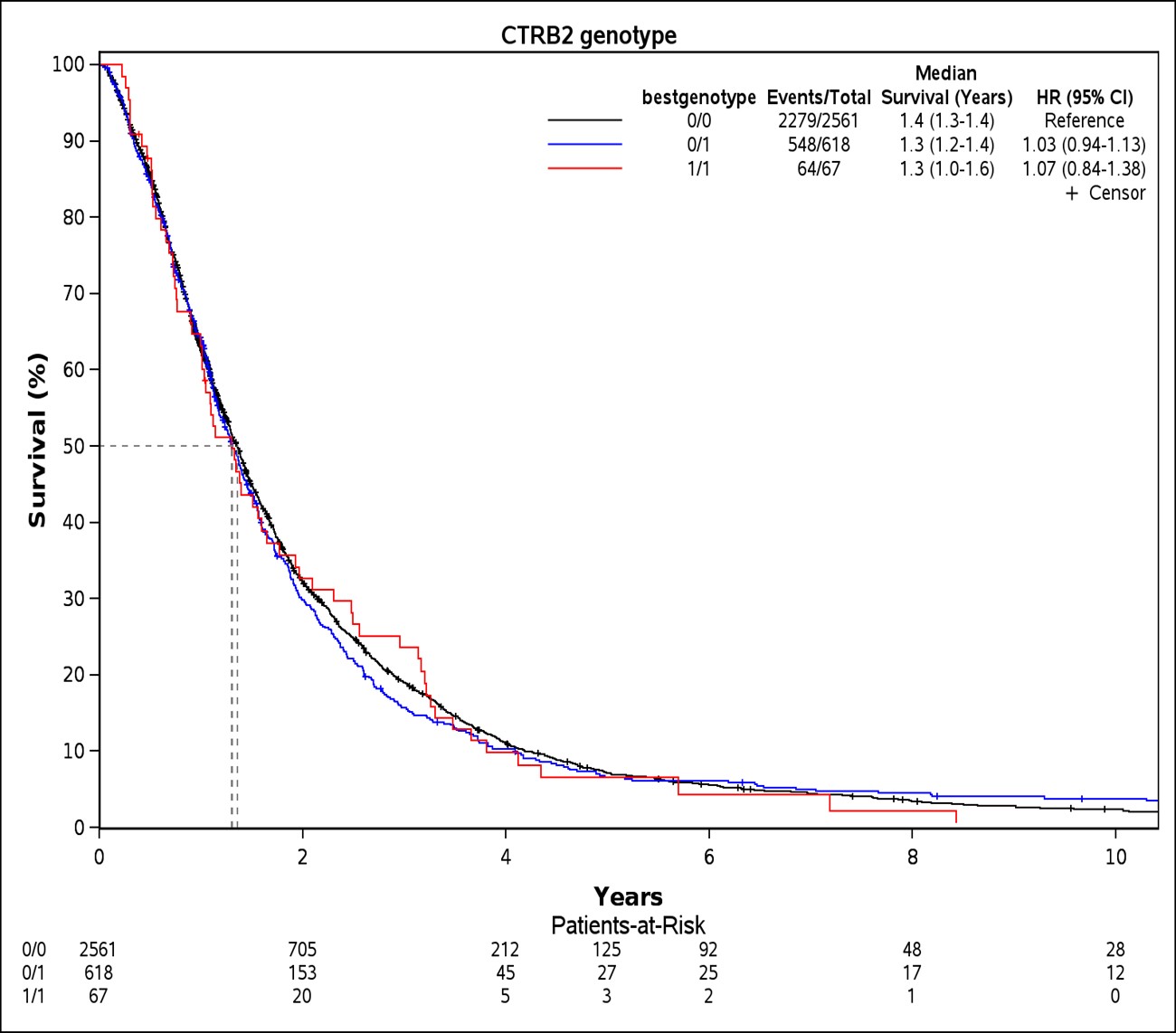

Supplement: S2 Fig — Cohort 2 comprises pancreatic ductal adenocarcinoma patients included in a genotyping by sequencing project performed in collaboration with the Regeneron Genetics Center (n = 3246 after removing patients with missing information). (JPEG) [file pone.0343022.s003.jpeg]

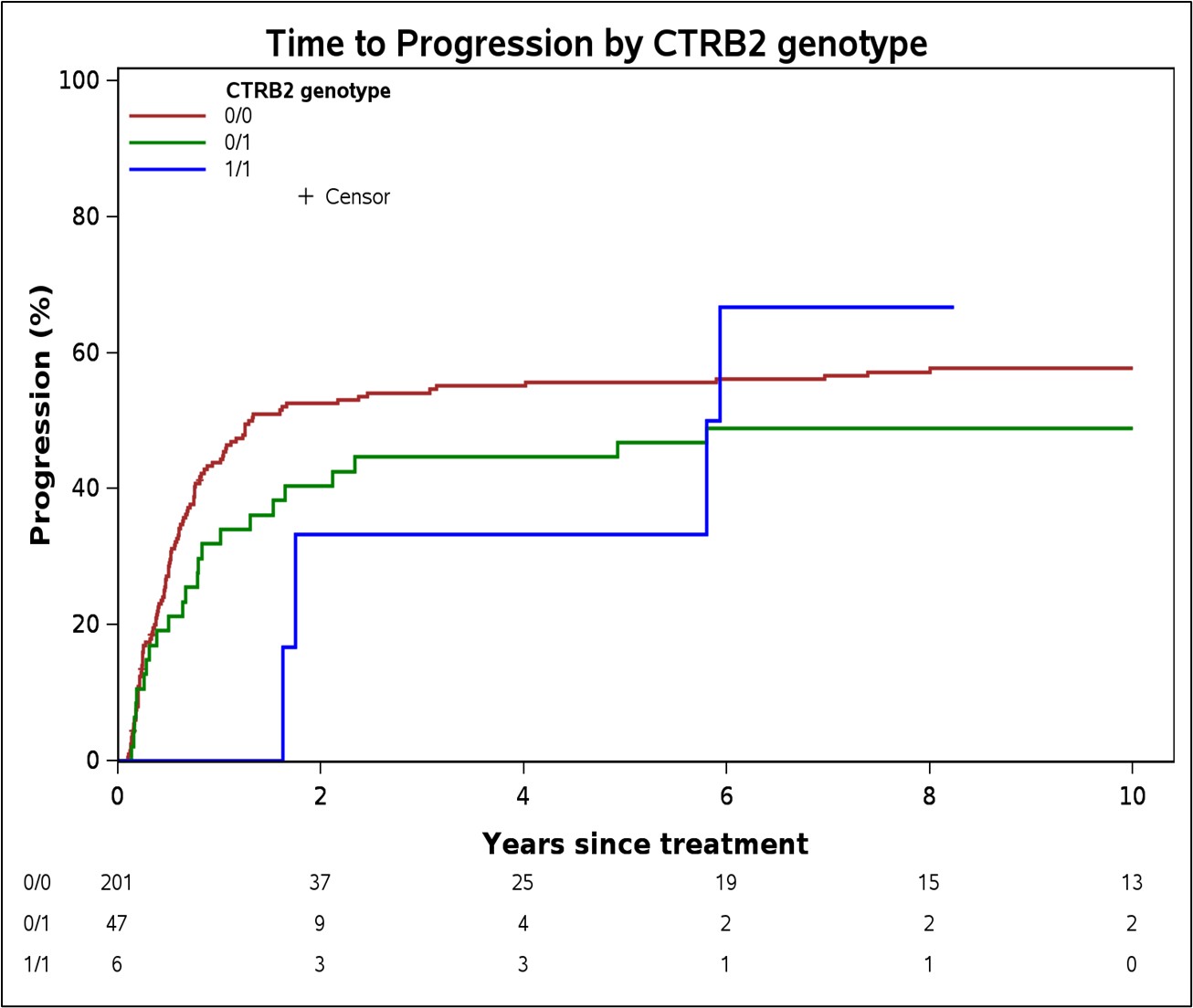

Supplement: S3 Fig — Cohort 1 comprises pancreatic ductal adenocarcinoma patients included in previous genome-wide association studies conducted in collaboration with the Pancreatic Cancer Cohort Consortium (PanScan) and the Pancreatic Cancer Case Control (PanC4) consortia (n = 254 after removing patients with missing information). (JPEG) [file pone.0343022.s004.jpeg]
