# Supplementary material for: Alterations in the CTRB2 gene and response to chemotherapy in pancreatic cancer
Source: PLoS One. 2026 Feb 19;21(2):e0343022. doi: 10.1371/journal.pone.0343022 (PMC12919833; doi:10.1371/journal.pone.0343022)
Supplement: S1 Table — (DOCX) [file pone.0343022.s005.docx]

| **Suppl. Table 1**. Association between CTRB2ex6 deletion status and time to progression (TTP) by treatment type among patients with PDAC | | | | | | | | |
| --- | --- | --- | --- | --- | --- | --- | --- | --- |
|  | 5-fluorouracil-based chemotherapy  (N=103) | | | | Gemcitabine  (N=73) | | | |
| CTRB2ex6 deletion | N | Median TTP (95% CI) months | HR (95% CI)^a^ | *P* | N | Median TTP (95% CI) months | HR (95% CI)^a^ | *P* |
| Absent | 80 | 15.6 (10.8-37.2) | 1.00 (ref) |  | 56 | 14.4 (9.6-NE) | 1.00 (ref) |  |
| Present | 23 | 58.8 (20.4-NE) | 0.66 (0.35-1.27) | 0.20 | 17 | 9.6 (6.0-NE) | 0.80 (0.33-1.97) | 0.63 |
| ^a^Adjusted for age, sex, and diabetes status.  Abbreviations: CI, confidence interval; HR, hazard ratio; OS, overall survival; PDAC, pancreatic ductal adenocarcinoma; TTP, time to progression | | | | | | | | |
